# Modelling microbial communities: Harnessing consortia for biotechnological applications

**DOI:** 10.1016/j.csbj.2021.06.048

**Published:** 2021-07-03

**Authors:** Maziya Ibrahim, Lavanya Raajaraam, Karthik Raman

**Affiliations:** a Bhupat and Jyoti Mehta School of Biosciences, Department of Biotechnology, Indian Institute of Technology (IIT) Madras, Chennai 600 036, India; b Centre for Integrative Biology and Systems Medicine (IBSE), IIT Madras, Chennai 600 036, India; c Robert Bosch Centre for Data Science and Artificial Intelligence (RBCDSAI), IIT Madras, Chennai 600 036, India

**Keywords:** Metabolic modelling, Genome-scale models, Metabolic engineering, Constraint-based modelling, Microbiome, Microbial consortia

## Abstract

Microbes propagate and thrive in complex communities, and there are many benefits to studying and engineering microbial communities instead of single strains. Microbial communities are being increasingly leveraged in biotechnological applications, as they present significant advantages such as the division of labour and improved substrate utilisation. Nevertheless, they also present some interesting challenges to surmount for the design of efficient biotechnological processes. In this review, we discuss key principles of microbial interactions, followed by a deep dive into genome-scale metabolic models, focussing on a vast repertoire of constraint-based modelling methods that enable us to characterise and understand the metabolic capabilities of microbial communities. Complementary approaches to model microbial communities, such as those based on graph theory, are also briefly discussed. Taken together, these methods provide rich insights into the interactions between microbes and how they influence microbial community productivity. We finally overview approaches that allow us to generate and test numerous synthetic community compositions, followed by tools and methodologies that can predict effective genetic interventions to further improve the productivity of communities. With impending advancements in high-throughput omics of microbial communities, the stage is set for the rapid expansion of microbial community engineering, with a significant impact on biotechnological processes.

## Graphical abstract

**Figure:**
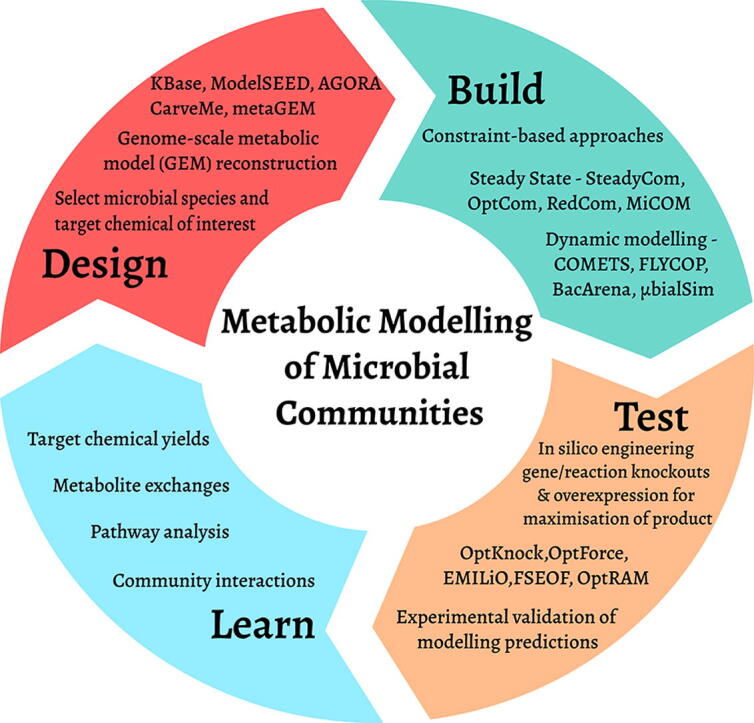

## 1 Introduction

The production of high-value industrial chemicals from renewable resources such as non-food biomass using microorganisms is an important pillar of sustainable biotechnology. Biotechnological processes involving microbial fermentations have become increasingly popular, replacing many chemical syntheses [1]. The choice of microbial host for the production of bio-based chemicals depends on whether: (i) the microbe naturally overproduces the target chemical, or (ii) the microbe produces the chemical but with low efficiency, or (iii) the microbe does not natively produce the chemical at all [2]. Each microbial host is then subjected to varied metabolic engineering strategies, depending on the three cases. Microbial “cell factories” of species belonging to diverse genera, such as *Escherichia, Clostridia, Saccharomyces, Corynebacterium*, *Bacillus,* and the fungi *Aspergillus* have been exploited owing to their better substrate utilisation and greater production capabilities [3], [4], [5], [6]. Compounds that are routinely synthesised by such microbes include 1,3-propanediol, ethanol, butanediol, succinate, malate, acetate, polyhydroxyalkanoates, fatty acid derivatives, amino acids such as L-lysine, citrate, and terpenoids [2].

Although engineered single microbial species have shown phenomenal success in the efficient production of chemicals, the metabolic capacity of single strains is still limited. In some cases, the yield and productivity are not high enough for expanding it to the industrial scale [7]. This is mainly due to the limitation in the number of genetic manipulations that can be implemented. Each intervention increases metabolic stress, which reduces the growth rate of the organism [8]. Hence, the incorporation of complex biosynthetic pathways is challenging. Similarly, in the case of substrates such as lignocellulosic biomass, which comprise an array of carbon sources, all the constituents cannot be utilised by a single organism [9]. Engineering monocultures to utilise multiple carbon sources results in a significant drop in efficiency [10].

Many of the challenges encountered when using monocultures can be circumvented by employing microbial communities. Communities possess beneficial attributes such as modularity, robustness to perturbation, and efficient task allocation [11]. The total metabolic capability of a community is often greater than the sum of the metabolic capabilities of its constituent members [12], [13]. This increased biosynthetic capability occurs in communities where the members are phylogenetically neither too close nor too distant and can be observed under two conditions: first, when the two organisms are initially introduced, and second, when the nutrients in the medium are exhausted [14]. Given the ability of microbial consortia to adapt to changing environments and robustly perform various tasks, their potential can be harnessed for metabolic engineering.

In this review, we discuss the key features that render microbial communities appealing for biotechnological applications. Beginning with the rules underlying community assembly, we catalogue some examples of successful exploitation of microbial communities for bioprocesses. We provide a broad overview of the existing computational tools and algorithms that equip us to build, study and analyse microbial communities. Finally, we cover the methods available to efficiently design and engineer microbial communities to produce any chosen metabolite of interest. We conclude our review by discussing key challenges in this field and the immense promise it holds for the future.

## 2 Microbial communities: rules of engagement

Microbial communities have intrinsic properties that can help us design better bioprocesses and also produce specific products that are otherwise very difficult to produce using monocultures [15]. Natural microbial consortia digest several carbon sources and are robust to environmental perturbations [16], making them viable candidates for processes that require the utilisation of multiple substrates [17]. Communities have been successfully adopted for the consolidated bioprocessing of lignocellulose, where the various components of the lignocellulosic biomass are broken down by different organisms [18]. Communities can also be chosen such that one or more members are the sole producers of the desired product molecule, while the others support them by breaking down multiple carbon sources and supplying the degraded substrates [19].

In communities, heterologous biosynthetic pathways are split into modules, and these sections are divided amongst the members. This reduces the metabolic stress the organism has to endure and distributes the workload [15]. This ‘division of labour’ is a quintessential feature of communities [20]. The synthesis of products is metabolically and spatially localised into modules in microbial communities [21]. This modularity improves the robustness of the system and can be helpful in cases where metabolic or spatial separation is required [22]. While organisms can share many simple metabolites and proteins [23], some non-native metabolites are accumulated within the cell, where the cell membrane of the organisms acts as a physical barrier for the upstream and downstream modules [24]. In such cases, the transfer of intermediate products from the upstream to the downstream module can be achieved by engineering transporters [25].

The organisms in a community share various metabolites that improve the growth and production capabilities of the community [26]. The excess metabolites produced by one organism are shared with the others [27]. This metabolite exchange reduces the accumulation of toxic metabolites and enhances the stress tolerance of the entire community [28]. It has been shown that the sharing of many metabolites does not have an associated fitness cost, i.e. they do not adversely affect the growth rate of the producer [29]. Organisms can also share electrons [30] and other molecules across longer distances through direct interspecies electron transfer (DIET) and nanotubes [31]. Metabolite exchange can also occur under nutritional stress [32], thereby promoting survival under environmental stress. This ‘network of support’ also stabilises the community and makes it robust to environmental perturbations.

### 2.1 Ecological interactions in microbial communities

**Fig. 1:**
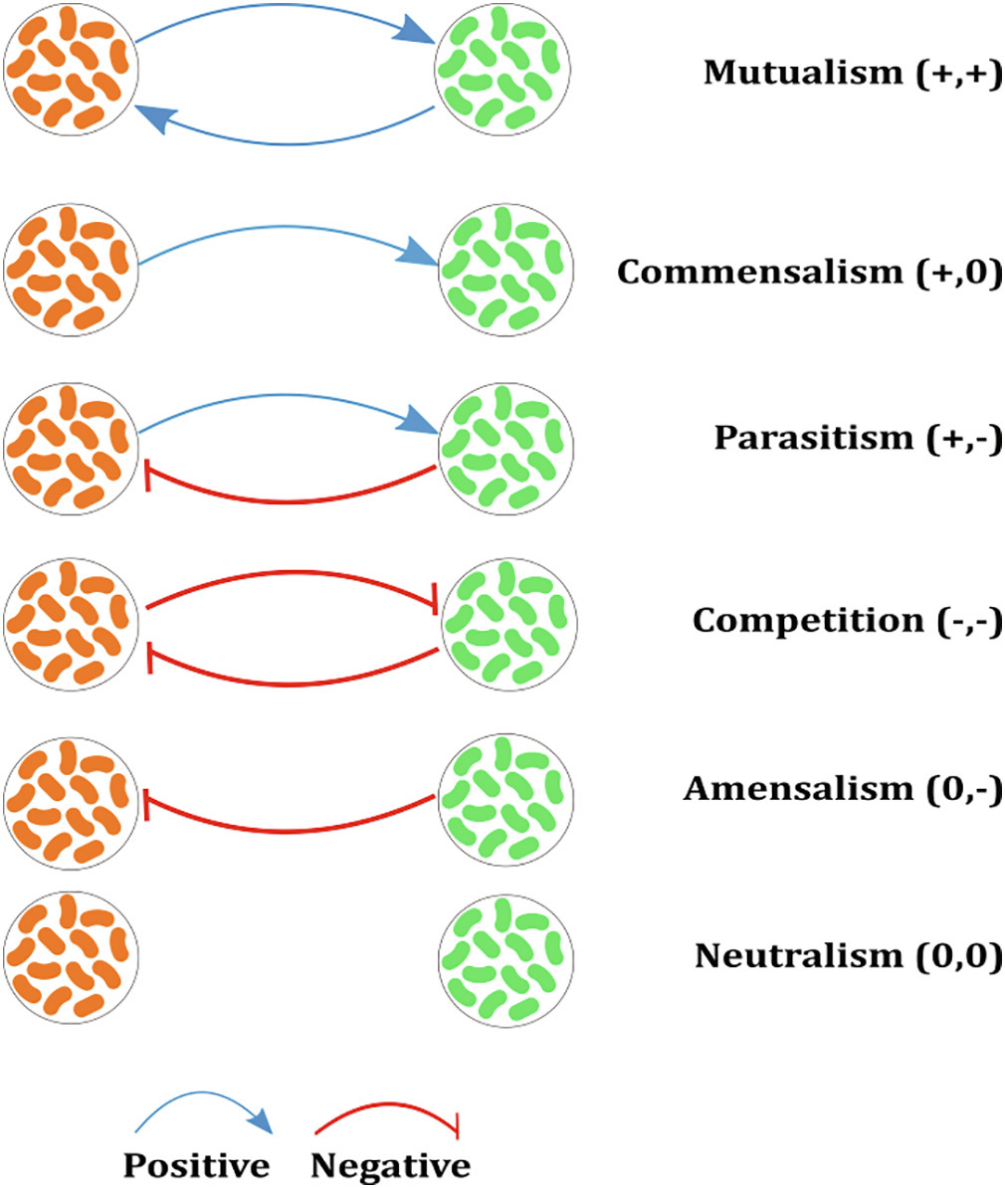
Types of interactions among two-member species of a community. (+) indicates a beneficial effect, while (−) indicates a detrimental effect, and (0) indicates a neutral, i.e. no effect.

There are various ecological interactions that occur in a community [33], [34]. These interactions can be broadly categorised as positive, negative, or neutral. They can also be termed as unidirectional or bidirectional interactions [35]. Mutualism or cooperative interactions, competition, and parasitic interactions are *bidirectional*, where both species under consideration are affected (Fig. 1). Commensalism and amensalism are *unidirectional* interactions, where only one partner is benefitted and negatively affected, respectively [35]. Kong *et al.* [36] have designed two-strain microbial consortia using *Lactococcus lactis* NZ9000 as host for each of the six types of social interactions. Using both experimental and modelling methods they showed that the consortia follow distinct population dynamics and that models derived from two-strain consortia can be used to design three or four-strain communities and predict their behaviour. Such social-interaction programming can be used to develop stability in the population and will improve the yield of chemical production during fermentation [36].

### 2.2 Classification of communities for the production of bio-based chemicals

**Fig. 2:**
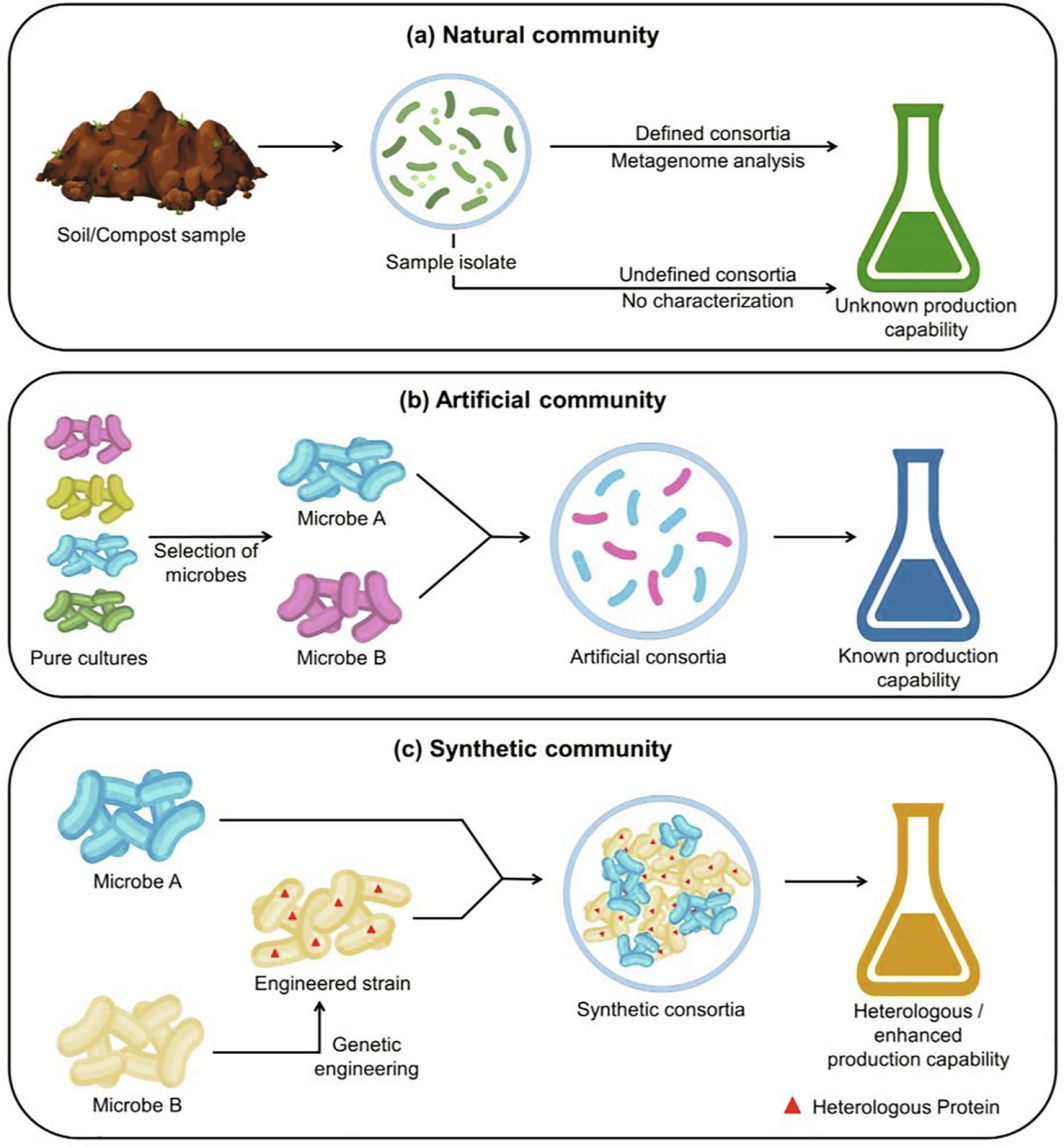
Types of microbial communities according to the nature of their assembly. (a) Natural consortia are isolated from nature for their collective metabolite production capability (b) Artificial consortia are assembled according to the favourable traits of their members (c) Synthetic consortia are engineered to improve the yield or to produce a metabolite that is not intrinsic to the community.

Microbial communities can be broadly classified based on the nature of their assembly. They can be isolated from natural environments (‘natural consortia’) or assembled artificially (‘artificial consortia’). The members of a community can also be engineered to improve their performance (‘synthetic consortia’). The community of interest can be chosen based on the needs of the bioprocess. These different types of communities are illustrated (Fig. 2) and discussed further in this section.

#### 2.2.1 Natural consortia

Microbial communities have co-evolved for ages to thrive in the environment surrounding them. They are well-adapted to share resources with their partners. These natural communities or consortia can be exploited to produce valuable metabolites. They can be isolated from various environments like soil, water, sludge, or compost according to the needs of the bioprocess. The members of such a community are usually not characterised and are called ‘undefined consortium’ [37]. In one study, an undefined mixed culture was isolated from cattle manure, cornfield soil, and rotten wood by selecting for its cellulolytic and butyrate-producing capability [38].

However, the identification of the members of the community can give us a better understanding and control of the metabolic interactions and community dynamics. The community can be characterised using metagenome sequencing, and the significant members can be cultured together to form a ‘defined consortium’ (Fig. 2a) [37]. Nevertheless, reduction of the number of members of the community has to be dealt with caution as the abundance of a member may not always be proportional to its significance in the community, and the loss of such significant members may cause loss of functionality or community instability [37]. A defined bacterial consortium (EMSD5) with high xylanase activity has been isolated from compost and used for consolidated bioprocessing of lignocellulose to produce isopropanol [39].

#### 2.2.2 Artificial consortia

Microbes can also be paired purely based on the process requirements — for their ability to breakdown specific substrates or to produce certain metabolites. These organisms may not coexist in the natural environment, but they are not metabolically engineered either. Such communities are termed ‘artificial consortia’ (Fig. 2b).

A thermophilic anaerobic co-culture of *Clostridium thermocellum* and *Clostridium thermolaticum* has been shown to exhibit up to a two-fold increase in ethanol yield when compared to either of the monocultures. This is attributed to better production of cellulases and xylanase by *C. thermocellum* and better utilisation of the degraded substrates by *C. thermolaticum* though both the strains can produce cellulolytic and xylanolytic enzymes [40]. Some organisms are chosen based on their ability to support the community by cross-feeding [41]. *S. cerevisiae* can produce large amounts of amino acids and secrete heat-shock proteins under environmental stress. These amino acids also enhance the alcohol tolerance of some anaerobic bacteria. Therefore, a co-culture of butanol-resistant *Clostridium beijerinckii* F-6 and *S. cerevisiae* was designed to improve butanol production [42]. The high oxygen sensitivity of *Clostridium beijerinckii* is a significant drawback of ABE (Acetone-Butanol-Ethanol) fermentation and this has been overcome by designing a co-culture of *C. beijerinckii* and *Bacillus cereus* which can grow under non-anaerobic conditions. The amylase activity of *B. cereus* further expands the range of substrate utilisation [28]. Communities also have an expanded metabolic space which is greater than the sum of the metabolic capabilities of the individuals [12]. A *C. acetobutylicum*–*C. ljungdahlii* co-culture has been shown to produce metabolites that are not produced by either of the monocultures [13]. This co-culture produces isopropanol as a result of the repurposing of a secondary alcohol dehydrogenase and the differential upregulation of the acetone pathway, which is in turn triggered by the direct cell-to-cell interaction and interspecies crosstalk. Competition in a co-culture of *Aspergillus fumigatus*–*Streptomyces peucetius* induces production of various alkaloids that are not produced by the monocultures [43]. Multiple homo- and hetero- cultures have been widely studied for their varied biosynthetic capabilities [5], [44]. Computational approaches have aided the study of complex artificial communities constituting as high as 25 microbes [45].

#### 2.2.3 Synthetic consortia

Microbes evolve in such a way that they can achieve maximum growth in the given environmental conditions. However, the goal of some bioprocesses may be to produce metabolites that are not growth-associated or even those that compete with growth. Therefore, it would be more desirable to *synthetically* engineer the microbes according to the specific needs of the bioprocess. This can increase the yield and efficiency of the process. Such engineered microbes form ‘synthetic consortia’ (Fig. 2c).

Co-cultures of phototrophic and fermentative bacteria have been shown to be promising for hydrogen production from lignocellulosic waste [46]. Such communities can be further optimised using metabolic engineering. In a co-culture of phototrophic *Rhodopseudomonas palustris* and fermentative *E. coli*, the mutant *R. palustris* NxΔAmtB can secrete more than three-times NH_4_^+^ than its wildtype strain. This enhances the growth of *E. coli* and thereby increases the rate of production of organic acids and their contribution to hydrogen production [47]. In another study, the biosynthetic pathway of resveratrol glucosides was split into two modules and expressed in two *E. coli* strains to reduce the metabolic burden. The first strain converted *p*-coumaric acid into resveratrol while the other converted it into its glucosides, polydatin, and resveratroloside [48].

The synthesis of heterologous products using microbial systems is gaining traction. However, the expression of complete pathways can cause a substantial metabolic burden to the organism. Flavonoids are high-value products that require pathway-engineering to be adapted to microbial systems. Upstream and downstream sections of the pathway have been successfully engineered in *E. coli,* but the incorporation of the complete pathway results in a remarkable decrease in overall yield. This has been circumvented by developing a co-culture of two *E. coli* strains, each containing the upstream and downstream modules separately. The process was further improved by optimising the temperature, carbon source, inoculation ratio, and induction point [15].

Competition for resources and natural selection can easily disrupt the community dynamics. *Corynebacterium glutamicum* has been widely used to produce various chemicals, but it cannot grow on starch. However, it can be co-cultured with an amylase-producing organism such as *E. coli* to utilise starch as a carbon source effectively. To prevent the outgrowth of *E. coli,* it can be engineered to be dependent on *C. glutamicum* for its survival. The *E. coli* has been engineered to be auxotrophic for L-lysine in this co-culture and has been optimised for cadaverine production [49]. Table 1 lists some microbial communities that have been exploited for biotechnological applications in recent years.

**Table 1:** Examples of products that have been successfully produced using microbial communities.

**Natural community**					
**Undefined community**					
	**Composition**	**Source**	**Product**	**Substrate**	**Ref**
1	Mesophilic microbes	Rumen fluid, Swamp and compost material	Carboxylic acids	Municipal solid waste, Sewage sludge	[50]
2	Anaerobic microbes	Activated sludge	Biogas	Food waste, cattle manure	[51]
3	Yeasts and bacteria	Activated sludge	Biopolymers	Crude glycerol	[52]
4	Hydrogen-producing bacteria	Anaerobic digested sludge	Hydrogen	Glucose	[53]
5	Anaerobic bacteria and fungi	Cow Manure	Hydrogen	Cellulose	[46]

**Defined community**					

	**Predominant organisms**	**Source**	**Product**	**Substrate**	**Ref**
1	*Clostridia*, *Actinobaculum*, *Pseudomonas*, *Azotobacter* and *Bacillus*	Marine sediment	Carboxylic acids	Sorghum	[54]
2	*Bacteroidetes*, *Firmicutes*, *Proteobacteria*, *Methanoculleus* and *Methanomassiliicoccus*	Pig manure	Biogas	Corn stover	[55]
3	*Clostridium*, *Escherichia*, *Bacillus*, *Lysinibacillus*, and *Firmicutes*	Compost soil	Isopropanol	Corncob	[38]
4	*Clostridium*, *Pseudomonas*, *Desulfovibrio*, *Bacteroides*, *Petrimonas*, *Escherichia*, *Shigella* and *Alistipes*	Goat or sheep faeces	Caproic acid	Ethanol and acetic acid	[56]
5	*Proteobacteria*, *Bacteroidetes*, *Actinobacteria*, *Chloroflexi*, and *Verrucomicrobia* and *Firmicutes*	Soil	Butyrate, Hexanoate, Octanoate	Acetate and ethanol	[57]

**Artificial community**							
	**Organism 1**	**Organism 2**	**Other microbes**	**Role in the community**	**Product**	**Substrate**	**Ref**
1	*Lactobacillus kefiranofaciens*	*Saccharomyces cerevisiae*	NA	Lk – produces kefiran; Sc – lactic acid consumption thereby improving tolerance	Kefiran	MRS medium	[58]
2	*Aspergillus fumigatus*	*Streptomyces peucetius*	NA	Not clearly understood	*N*-Formyl Alkaloids	International Streptomyces Project Medium 2 (ISP2)	[43]
3	*Bacillus cereus*	*Clostridium beijerinckii*	NA	Bc – utilises starch; Cb – produces butanol	Butanol	Corn mash	[28]
4	*Penicillium fuscum*	*Penicillium camembertii/ clavigerum*	NA	Not clearly understood	Berkeleylactones, Antibiotic macrolides	Potato dextrose broth	[59]
5	*Trichoderma reesei*	*Rhizopus delemar*	NA	Tr – breakdown cellulose; Rd – produces fumaric acid	Fumaric acid	Corn stover	[44]
6	*Trichoderma reesei*	*Rhizopus oryzae*	NA	Tr – breakdown cellulose; Ro – produces lactic acid	Lactic acid	Microcrystalline cellulose	[44]
7	*Ralstonia eutropha*	*Bacillus subtilis*	NA	Re – produces PHA; Bs – sucrose utilisation	P(3HB-co-3HV) polymer	M9 minimal medium with sucrose	[19]
8	*Clostridium beijerinckii*	*Saccharomyces cerevisiae*	NA	Cb – produces butanol; Sc – produces amino acids for Cb and improves alcohol tolerance	Butanol	Glucose	[42]
9	*Clostridium beijerinckii*	*Yokenella regensburgei*	NA	Cb – produces hydrogen; Yr – produces lactate which boosts growth and hydrogen production by Cb	Hydrogen	Food waste	[60]
10	*Schizophyllum commune*	*Bjerkandera adusta*	*Fomitopsis palustris*	Sc – ethanol production; Ba – lignin degradation; Fp – cellulose degradation (all three microbes produces ethanol)	Ethanol	Japanese cedar wood chips	[61]
11	*Bacteroides vulgatus*	*Desulfovibrio piger*	*Bifidobacterium longum*, *Eubacterium rectale*, *Roseburia intestinalis* + 20 microbes	Not clearly understood	*N*-Formyl Alkaloids	DM38 medium	[45]

**Synthetic community**							
	**Organism 1**	**Organism 2**	**Role in the community**	**Significant mutations**	**Product**	**Substrate**	**Ref**
1	*Escherichia coli*	*Escherichia coli*	Ec1 – produces *p*-coumaric acid; Ec2 – converts *p*-coumaric acid to caffeyl alcohol and coniferyl alcohol	*E. coli* ATCC 31884 with *pheA* and *tyrA* disrupted, cloning of various plasmids with genes that encode for *p*-coumaric acid production	*p*-coumaryl alcohol, caffeyl alcohol and coniferyl alcohol	Modified M9 (M9Y) medium	[62]
2	*Synechococcus elongatus*	*Halomonas boliviensis*	Se – photosynthetically fixes carbon and exports as sucrose; Hb – produces PHB	Se – cloning of *cscB* gene, a sucrose transporter	Polyhyroxy-butyrate	Carbon dioxide	[63]
3	*Escherichia coli*	*Corynebacterium glutamicum*	Ec – utilisation of starch; Cg – production of L-lysine	Ec – deletion of *lysA* to make it a lysine-auxotroph and cloning of EcLys1 (α-amylase) from *S. griseus* to utilise starch; Cg – multiple deletions (Δ*pta-ackA* Δ*cat* Δ*aceAB* Δ*ldhA* Δ*nanR*) to get strain CgLys4 for better production of L-lysine	L-lysine, L-pipecolic acid, cadaverine	Starch and sucrose	[49]
4	*Escherichia coli*	*Escherichia coli*	Ec1 – converts *p*-coumaric acid into resveratrol; Ec2 – converts the resveratrol into polydatin and resveratroloside	Ec1 – *E. coli* BL21 (DE3) containing pAC-4CL-STS (Cm); Ec2 – *E. coli* BL21 (DE3)/Δ*pgi*/Δ*zwf* containing pET28a-hasC (Km) and pQE30-PaGT3 (Amp)	Resveratrol	M9 minimal medium with glucose	[48]
5	*Escherichia coli*	*Saccharomyces cerevisiae*	Ec – produces ethanol from xylose; Sc – produces ethanol from glucose	Ec – deletion of *ptsG*, *pgi* and *zwf* genes to construct glucose negative strain *of E. coli* strain SL100	Ethanol	Sugar cane bagasse	[64]
6	*Pichia pastoris*	*Pichia pastoris*	Pp1 – converts to dihydromonacolin L (DML); Pp2 – converts DML to monacolin and lovastatin	Pp1 – cloning of *lovB*, *lovC*, *lovG*, *npgA* genes; Pp2 – cloning of *slovA*, *cpr* genes	Monacolin and lovastatin	Methanol	[65]
7	*Escherichia coli*	*Rhodopseudomonas palustris*	Ec – produce organic acids; Rp – convert organic acids to hydrogen	Rp – deletion of *nifA*, *amtB1*, *amtB2* genes for NH^4+^ excretion, deletion of *hupS* to prevent H_2_ oxidation and *uppE* to prevent biofilm formation	Hydrogen	M9-derived coculture (MDC) medium	[47]
8	*Escherichia coli*	*Streptomyces venezuelae*	Ec – produces phenylpropanoids like pterostilbene, naringenin, and apigenin; Sv – expresses a methyltransferase that catalyses mono-, di-, and tri-methylation of phenylpropanoids	Ec – cloning multiple genes responsible for phenylpropanoid synthesis (Os4CL, VvSTS, VvROMT, PeCHS, PcFNS and MtCHI); Sv – deletion of pikromycin polyketide synthase and cloning of a methyltransferase from *Streptomyces avermitilis* (SaOMT2)	*O*-methylated phenylpropanoids	LB medium and R2YE medium	[66]
9	*Klebsiella pneumonia*	*Shewanella oneidensis*	Kp – converts glucose and xylose into lactate; So – electron donor	Kp – deletion of *adhE*, *pta* genes and cloning of *ldhD*, *lldP* genes to improve lactate production; So – deletion of 1S1 and cloning of *ribA*, *ribD*, *ribE* and *ribH* genes to improve direct-contact extracellular electron transfer	Electricity	Corn stalk hydrolysate	[67]
10	*Clostridium beijerinckii*	*Clostridium cellulovorans*	Cb – produces butanol; Cc – breakdown lignocellulose	Cc – deletion of cell wall lyases genes (Clocel_0798 and Clocel_2169) and overexpression of agmatine deiminase genes (*augA*, encoded by Cbei_1922) from *C. beijerinckii* to improve pH tolerance, cloning of gene *adhE1* from *Clostridium acetobutylicum* for butanol production	Butanol	Glucose	[68]

## 3 Modelling microbial communities

**Fig. 3:**
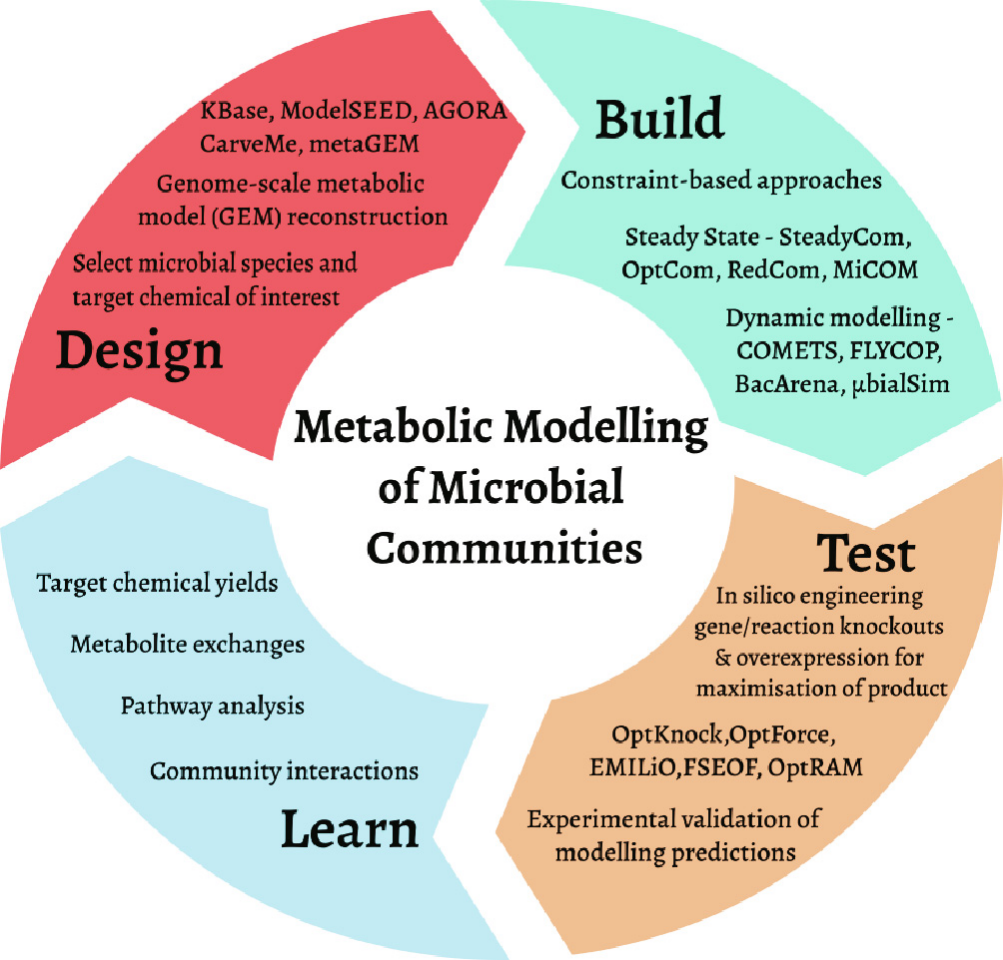
A Design–Build–Test–Learn cycle for metabolic modelling of microbial communities. The figure gives an overview of the topics discussed in this review. The ‘design’ stage includes the selection of the target chemical of interest and the microbial species that can produce the chemical. GEMs are reconstructed for the selected microbial species. In the ‘build’ stage, community models are generated using GEMs of single species. Both steady-state and dynamic based methods are available. The ‘test’ phase allows improvements in the metabolic capabilities of the microbial community through *in silico* gene knock-out or overexpression. The computational predictions are then tested experimentally, helping us to ‘learn’ about the community interaction behaviour, identify efficient pathways and the maximum product yield achievable by the chosen community.

Metabolic interactions are the key drivers of many microbial communities [69]; therefore, modelling and understanding microbial metabolism can provide significant insights into how microbes assemble into communities, how they interact, and how they can be leveraged for biotechnological processes. Over the last few decades, several methods have been developed to model microbial metabolism [70], [71], and some of these have also been extended to model microbial communities [37], [72], [73]. While a variety of approaches exist, the most popular strategies for modelling microbial metabolism are based on the paradigm of constraint-based modelling (Fig. 3).

Constraint-based modelling is an approach that can be applied to any biological system at the level of either single organisms or multi-species consortia [74]. The modelling begins with gene annotation of genomic data that aids in the reconstruction of a draft genome-scale metabolic model (GEM). This is followed by several stages of model refinement, including “gap-filling” [75], which aims to fully connect the metabolic network and result in a more complete model, ready for analyses. Experimental data can be integrated into such models to further enhance their predictive power [76].

One of the earliest attempts at modelling microbial communities was adopted by Stolyar and co-workers, who investigated the co-culture of *Desulfovibrio vulgaris* and *Methanococcus maripaludis* [77]. They demonstrated the use of genome-scale metabolic models (GEMs) to capture community growth parameters. Ye *et al.* designed an artificial community of *Ketogulonicigenium vulgare* and *Bacillus megaterium* to produce vitamin C [78]. They utilised GEMs to understand the inter-species interactions in the co-culture. Below, we discuss some of the key methods, starting with the reconstruction of these GEMs, methods for their simulation, and most importantly, how these methods are extended for modelling microbial communities. We also briefly discuss other methods that have been broadly used to model microbial communities but have not yet found significant application in metabolic engineering.

### 3.1 Genome-scale metabolic models (GEMs) and constraint-based modelling approaches

Genome-scale Metabolic models (GEMs) computationally and mathematically represent the metabolism of a species. Several such GEMs have been reconstructed for thousands of organisms over the last two decades [79]. These metabolic reconstructions capture all known reactions in a cell through stoichiometric matrices. Gene–Protein–Reaction (GPR) associations in these reconstructions further describe how enzymes in the organism catalyse the various reactions, capturing the many-to-many relationship between genes, proteins and reactions in the cell [80]. GEMs are typically used to predict various phenotypes through the optimisation of an objective function, and have a variety of applications [81].

The first GEM was developed for *Haemophilus influenzae* [82]. Since then, the number of metabolic reconstructions and the tools to develop and analyse such models has significantly increased [79]. GEMs have been applied for strain development of microbes to produce high-value chemicals, prediction of enzyme functions, and modelling of microbial communities [83], [84], [85], [86].

GEMs can be easily reconstructed using the genome sequences and the metabolic reaction information from knowledgebases such as KEGG [87] and MetaCyc [88]. Studies such as Path2Models [89], AGORA [90], and CarveMe [91] have made more than 6000 metabolic models available. These models span various domains, including Archaea, Eukarya, and Bacteria. Some representative GEM databases are BiGG Models [92] (http://bigg.ucsd.edu), which contains over 100 manually reconstructed high-quality GEMs, and BioModels [93] (https://www.ebi.ac.uk/biomodels/), which is a repository of both curated, non-curated models.

To keep pace with the surge in genome sequencing, numerous approaches have also been developed to automate the reconstruction of GEMs [76], [94]. ModelSEED [95] (http://modelseed.org) was the first web-based reconstruction tool to enable automated reconstruction of GEMs. KBase [96] (http://kbase.us) is another open-source software and data platform that hosts a suite of tools that facilitates RNAseq read alignment and genome annotation, including the reconstruction and analysis of microbes and their communities. Recent tools include RAVEN [97] and AutoKEGGRec [98], both of which use KEGG database for reconstruction. AuReMe [99] is an adaptable workspace with enhanced traceability that uses a template-based algorithm and can incorporate information from multiple databases.

In some cases, the reconstructed models primarily represent reactions from the central metabolic pathways. Incorrect or missing functional annotation of genes in databases can lead to the reconstruction of metabolic models that have gaps in pathways and do not capture accurate strain-specific metabolic capabilities [100]. Gapseq is a recently published automated pipeline that attempts to address this by using a novel gap-filling method to decrease the effects of arbitrary growth medium measurements [101]. The tool also includes phenotypic data from non-model organisms to validate the metabolic reconstructions, thus making this a robust approach with a greater true positive rate for enzymatic activity, carbon source utilisation and fermentation products.

All the above approaches, to some extent, depend on manual curation to guarantee the quality and accuracy of the model. Manual curation tasks include detection of futile cycles, removal of blocked reactions and checking the directionality of reactions in the model [80].

GEMs can also be generated for metagenome-assembled genomes (MAGs) with new automated methods such as metaGEM [102]. Such “draft” reconstructions are very helpful in making a first-cut prediction of the metabolic capabilities of an organism/community. Nevertheless, it is crucial to further accurately curate the model, to improve its quality and reliability. Helpful in this regard are tools such as MEMOTE, which is a suite of tests for the metabolic model used to assess its quality and ensure reproducibility of results [103]. This is especially important for microbial community simulations as insights derived from these analyses can be biased with sub-standard GEMs.

Other inherent challenges specific to metagenomic data include pre-processing of abundance data which can arise due to differences in sequencing methodologies. Also, there is a lack of a ‘gold standard’ where information of community interactions and sequencing data are known [104]. Nevertheless, imminent advances in this area can not only improve the pace of reconstruction and analysis, but also ensure reproducibility through consistent protocols.

#### 3.1.1 Stoichiometric matrices – single-species GEMs and community GEMs

A defining mathematical object of every GEM is the stoichiometric matrix $S_{m\times n}$**,** where the rows represent the m metabolites, and the columns correspond to the n reactions. Each entry in the matrix represents the stoichiometric coefficient of a particular metabolite in a reaction. This mathematical representation of metabolic reactions is one of the first steps in FBA. The flux through all the reactions is given by the vector $v_{n\times 1}$, and the concentrations of the metabolites are represented by vector $x_{m\times 1}$. At steady state, S·ν=0, giving rise to a system of linear equations, which is typically under-determined. Thus, to pick a biologically relevant solution, an objective function is employed. Linear objective functions are most widely used, given as, $Z=c^{T}v$, where **c** is a vector of weights specifying the contribution of each reaction to the objective function [105].

A community GEM is considered as a multi-compartment model where a community compartment typically denoted as ‘[u]’ allows for the exchange of metabolites between the species. The stoichiometric matrix of a microbial community model consisting of two species can be represented as $S_{M\times N}$. The columns represent the total number of reactions ***N*** (*n*_1_ + *n*_2_ + *n*_e_) from species 1 (n_1_), species 2 (n_2_), and the community exchange reactions (n_e_). The rows represent the total metabolites ***M*** (*m*_1_ + *m*_2_ + *m*_e_) from species 1 (m_1_), species 2 (m_2_), and the metabolites that are part of the community exchange reactions (m_e_).

**Fig. 4:**
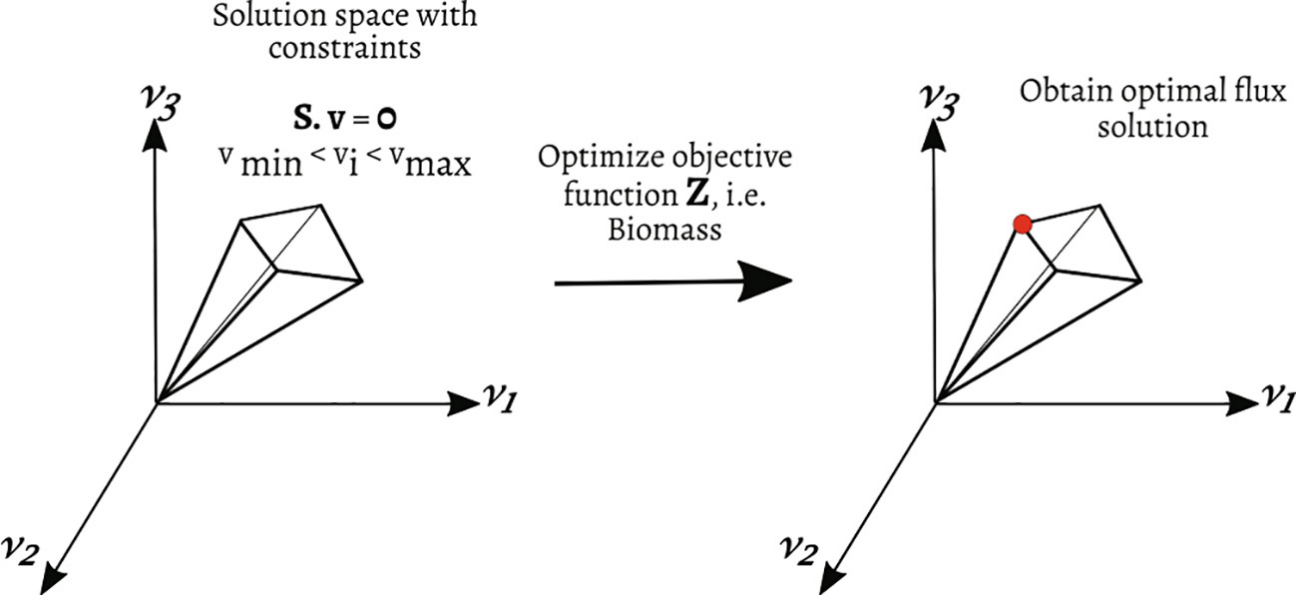
A schematic of constraint-based modelling using flux balance analysis (FBA). The allowable solution space, which contains all possible flux distributions, is constrained by mass balance, thermodynamics, and capacity constraints. The red dot on the edge of the solution space represents an optimal flux solution for the specific objective function Z. (For interpretation of the references to colour in this figure legend, the reader is referred to the web version of this article.)

Constraint-based modelling has been used to assess and interpret GEMs. Fluxes of the metabolic network are subjected to various physicochemical constraints that are driven by culture conditions, thermodynamics, and steady-state assumptions. FBA calculates metabolic fluxes of all reactions in a GEM through a linear programming-based optimisation of a biologically relevant objective function (Fig. 4). The objective functions could be maximising the biomass formation rate or the target product formation rate [105], [106].

Even for a given objective function, FBA may predict multiple alternative optimal solutions. This limitation of FBA can be somewhat overcome by Flux Variability Analysis (FVA) and parsimonious FBA (pFBA) methods. FVA identifies the flux range (minimum and maximum) of all the reactions in the metabolic network [107]. pFBA computes a flux distribution that minimises the sum of all fluxes in the model while satisfying a particular objective [108].

### 3.2 Computational tools to build constraint-based community models

Many computational tools based on constraint-based modelling have been developed to design microbial communities [109], [110]. These methods vary in their assumptions of steady-state growth, the choice of the objective function, and also in their treatment of spatial distributions of the communities. Table 2 contains a summary of the widely used constraint-based computational methods for modelling.

**Table 2:** Constraint-based computational tools for modelling microbial communities.

**Computational Methods**	**Input data**	**Community Size**	**Objective Function**	**Predictions**
***Steady-state modelling***				
cFBA	GEMs	Small	Maximisation of community growth rate	Predicts species abundances and identifies cross-feeding metabolites
OptCom	GEMs	Small	Multi-objective optimisation, where the inner problem is maximisation of species-level growth and the outer problem is maximisation of community growth	Predicts inter-species metabolite transfers
MMinte	Operational Taxonomic units (OTUs) & FASTA file with 16S rDNA sequences	Large	Maximisation of community growth rate	Reconstructs metabolic models and predicts growth rate Generates interaction networks
SteadyCom	GEMs	Large	Maximisation of community growth rate	Predict composition (species abundances) of microbial community in a given environment
RedCom	Elementary Flux Vectors (EFVs)	Large	Maximisation of community growth rate	Predicts feasible ranges for metabolite exchange rates and product yields
Microbiome Modelling Toolbox (MMT)	GEMs and microbial Metagenomic data	Large	Maximisation of community growth rate	Predicts metabolic profiles in pairwise as well as larger microbial communities
CarveMe	Genome FASTA files	Large	Maximisation of community growth rate	Reconstruction and gap-filling of single-species metabolic models. Generate microbial community models from single species

***Dynamic modelling***				
BacArena	GEMs	Greater than 2 species	Individual-based modelling with FBA where Biomass maximisation is objective	Predict cross-feeding interactions Metabolic turn over using metabolite concentrations as constraints
COMETS	GEMs, media and spatial structure simulation parameters such as *mutRate* that represent mutations	Greater than 2 species	Population based-modelling where maximisation of biomass is the objective	Outputs can be from all or selected time steps. Predicts biomass spatial distribution for each simulation grid cell. Tracks specific metabolites on the spatial grid
*µ*bialSim	GEMs	Large	Dynamic FBA, both batch and chemostat operations are simulated	Simulation of microbiomes, where metabolite exchange is the primary means of interaction
FLYCOP	GEMs	Small	Multiple objectives such as maximise growth, yield, metabolite production, minimise time to reach stationary phase etc	Predict ideal consortium configuration depending on the optimisation goal

**cFBA**[111] models the metabolic behaviour of communities at steady-state. To maintain the steady-state, all the members of the community should have the same specific growth rate while the changes in the exchange rates should be proportional to that of biomass, or the species abundance must remain constant with null net growth rate. This method is limited to communities with balanced growth and is computationally very expensive for large communities. These drawbacks have been overcome to some extent by the tools developed later.

**OptCom**[112] employs a multi-objective formulation to understand trade-offs between individual vs community-level fitness. The community-level objective function is the total community biomass, and an individual biomass maximisation problem is defined for each species as an inner problem. OptCom can also be customised for each type of interaction, such as mutualism, parasitism, and competition, by altering the inter-organism flow constraints. This algorithm was used to predict acetate and CO_2_ production rates in a community comprised of *Clostridium cellulolyticum*, *D. vulgaris,* and *G. sulfurreducens.* OptCom can be used to identify minimal number of knock-outs required to result in higher production of a desired compound by setting the objective function of the outer problem as production of the compound of interest [112].

**MMinte**[113] is a pipeline that allows the comparison of growth rates between pairs of members in a community. It uses 16S rDNA sequence data to identify reference genomes and reconstructs GEMs. The application consists of seven widgets with different functionalities that can be tested individually or sequentially. A colour-coded interaction network based on the community interactions can also be visualised. MMinte assigns functional interactions between members of a community instead of only computing correlations based on microbial abundance. MMinte has been used to observe the influence of diet on the type of microbial interactions. It has also been used to understand the growth patterns of *Desulfovibrio piger* in an eight-member gut community of gnotobiotic mice. Limitations of MMinte are related to the accuracy of the metabolic models generated, which uses reactions from ModelSEED. The method also does not account for the effect that other species in a community may have on the strength and type of interactions in a two-member community.

**SteadyCom**[114] is a constraint-based computational modelling framework for the generation and steady-state FBA of microbial communities. It identifies the relative abundance of each species with the objective function of maximisation of community growth. SteadyCom guarantees that in a growing community, the organism can have non-zero fluxes if and only if both the total biomass and the biomass production rate are non-zero. The algorithm is also compatible with FVA and is scalable to many organisms in a community. The computational time required by SteadyCom has been shown to be far less when compared to that of cFBA as the number of LPs required to solve SteadyCom is dependent only on the desired precision of the maximum growth rate. SteadyCom has been used to model a gut microbiota community consisting of nine species with dietary constraints to predict their relative abundance [114].

**RedCom**[115] is another method that proposes reduced community models where the metabolic reactions are represented as net conversions taken from elementary flux vectors of the various single-species networks. The predictive potential of such reduced communities is greater as they eliminate spurious solutions where one species produces a large number of compounds required by another species instead of synthesising its own biomass components. The drawbacks of this approach are that the internal flux distributions are not observed as the primary focus is on the exchange fluxes. RedCom has been used to model a nine-member community that is involved in anaerobic digestion in biogas plants. Metaproteomic data has also been used to constrain the solution space for the community models, which has helped identify acetoclastic methanogens from *Methanosarcinales* to be abundant in the community.

**Microbiome Modelling Toolbox**[116] is a MATLAB-based toolbox that can be used to construct and analyse microbial communities and host-microbe interactions. Personalised community models and pan-models can be created with functions that are part of the toolbox. To merge models, a uniform nomenclature of metabolites and reaction abbreviations is necessary. It has a pipeline known as *mgPipe,* which integrates microbial abundances from metagenomic data and metabolic reconstructions. It also contains built-in functions to determine the pairwise interactions for a community. The differences between uptake and secretion fluxes of each community model can also be analysed with multidimensional scaling methods.

**CarveMe**[91] is a Python package that can be used to construct both single-species and community models. It uses a top-down approach to construct the metabolic network of an organism from a universal model. This process can thus be easily parallelised. The universal model consists of a universal biomass equation, exchange reactions and does not have any blocked reactions. Multiple single-species models are merged to form a community model where each organism has its extracellular compartment connected to a shared resource pool. CarveMe relies on the BiGG database to build the universal model, and hence while primary metabolism pathways are essentially complete other secondary pathways will contain gaps and require further curation of the model.

It is often challenging to employ FBA methods to analyse the complex dynamics of microbial consortia. Therefore, to overcome the limitations posed by FBA, methods that model the temporal and spatial aspects of microbial systems have been developed, e.g., **dynamic FBA** (dFBA). dFBA is an extension of FBA that integrates the rate of change of flux constraints. Kinetic substrate uptake parameter values are also incorporated in dFBA analysis. dFBA applies to batch and fed-batch culture processes [117]. *S. cerevisiae* and *E. coli* co-cultures that can consume both glucose and xylose efficiently have been studied using dFBA to develop a community model that can account for the community growth conditions and interactions between the organisms [118]. Well-known modelling methods using principles of dFBA are discussed below.

**BacArena**[119] is an individual-based modelling approach. BacArena models communities as aggregates of individuals that have their distinct metabolism and interact with one another through spatial and temporal means that follow biological rules, i.e. through lysis, and chemotaxis. This can be used to hypothesize cross-feeding mechanisms between the species. BacArena has been applied to gain insights into the biofilm formation by *Pseudomonas aeruginosa,* and it has been identified that spatial gradients of mucus glycans were necessary for niche formations for a seven species community of the human gut.

**COMETS (Computation of Microbial Ecosystems in Time and Space)**[120] is another dynamic modelling method that differs from BacArena in the representation of the spatial scale. In COMETS a population of multiple cells are represented per grid position. COMETS can predict the growth rates according to the spatial concentration gradients. It has been used to study two and three-strain synthetic communities of *Salmonella enterica*, *Methylobacterium extorquens,* and *E. coli*. A recent version named COMETS 2 (https://www.runcomets.org/) includes new biological modules that encompass evolutionary dynamics and extracellular enzyme activity. It is compatible with both MATLAB and Python interfaces and COBRA models. Some limitations of COMETS arise from the complexity of numerical integration of the convection–diffusion equations, which requires the users to choose a small enough time step to prevent numerical errors. It is also not recommended to study phenotypic cell-to-cell variability in a population.

***µ*bialSim**[121] is a dFBA-based numerical simulator that can predict the time-course in terms of the composition and activity of microbiomes that contain numerous species either in batch or chemostat mode. Each species has access to a shared pool of metabolites that can be exchanged between the species. FBA simulations in *µ*bialSim can utilise functions from either COBRA Toolbox or CellNetAnalyzer. *µ*bialSim has been applied to a syntrophic methanogenic co-culture as well as a 773-species human gut microbiome [121]. This method can be used to explore microbial ecology principles such as substrate competition.

**FLYCOP** (**FL**exible s**Y**nthetic **C**onsortium **OP**timisation) [122] is a framework that analyses multiple microbial community configurations in an automated manner and selects the best configuration for a specific objective. GEMs of the microbial strains are given as input. This method utilises COMETS parameters for dynamic simulations. Different configurable consortium parameters include strain ratio, medium composition, cross-feeding rates, pathway fragmentation, and consortia partner selection. There is a limitation with respect to the computational time as it is dependent on the number of parameters to be configured. For the same reasons, FLYCOP simulations are restricted to a small number of species in the community.

Each of the computational modelling tools discussed above (also see Table 2) comes with its own pros and cons. Further, the number of microbial species that can be modelled using these methods can vary from a minimum of two microbial species (pairwise analysis) to large communities. Hence, a tool should be selected for use depending on the objective of the study and the size of the microbial community being analysed.

### 3.3 Alternate metabolic modelling approaches

Although constraint-based models are most popular, other models have also been developed, which provide complementary insights. In this section, we discuss alternate modelling approaches, such as those based on graph theory or on population modelling and agent-based modelling. For a more detailed account of these methods, see ref. [73], [123].

#### 3.3.1 Macromolecular expression (ME) models

Genome-scale models of metabolism and Macromolecular Expression or ME-models widen the scope of metabolic (M) models by incorporation of macromolecular biosynthesis pathways of transcription and translation. In ME-models, the metabolic reactions include substrate-enzyme binding and product-enzyme dissociation reactions. These models are also constrained by coupling constraints. Reactions catalysed by macromolecules are dependent on the synthesis of the macromolecule for the reactions to proceed. ME-models have greater accuracy than M−models as the flux solutions obtained are parsimonious [124]. COBRAme is a software framework built on COBRApy that can be used for building and simulating ME-models [125].

Probabilistic Regulation of Metabolism (PROM) computes probabilities retrieved from expression data to represent the gene states and transcription factor-gene interactions and constrain the fluxes through the network. ME-models can be expanded with such gene regulation data. Protein allocation may also be necessary for identifying the metabolic capabilities of microbial communities, and this gap can be filled by ME-models [124]. Recent progress has enabled the use of microbial community ME-models to study co-cultures growing in an adaptive laboratory evolution (ALE)-optimised experiment [126].

#### 3.3.2 Graph-based modelling of microbial communities

In graph-based modelling methods, GEMs are represented as graphs. Every graph G(V, E) is described by a set of nodes (or vertices, **V**), having connections to one another by means of the edges (**E**). ‘Substrate graphs’ connect metabolites with edges directed from substrates to products. The metabolites are denoted as nodes with edges representing the reactions to form substrate graphs. Alternatively, both metabolites and reactions can be represented as nodes, and the two types of nodes can be connected via edges to form a ‘*bipartite* graph’ [127]. Specific metabolites are defined as ‘seed’ sets, and these are consumed but not produced by the network. The seed metabolites are provided exogenously to the network, and the metabolic pathways are inferred using various pathfinding approaches [128], [129]. Such graph-based approaches help understand the evolutionary dynamics of competition or cooperation between species in a community [72]. MetQuest is another graph-theoretic algorithm that uses a guided breadth-first search to identify all feasible reactions based on the seed metabolite set [130]. These reactions are then assembled into pathways of different sizes that produce the target from the seed source. MetQuest has been employed to study all possible metabolic exchanges between *S. cerevisiae* and *Pichia stipitis* and understand how the two organisms benefit from each other in a co-culture. The findings concurred with observations seen in the co-culture experiments of the two species [131].

#### 3.3.3 Population-based modelling of microbial communities

Population models capture the population dynamics and the spatial distribution of the community [132]. Each community member is studied at the population level, and the system is modelled using ODE or PDE-based modelling. It also includes game-theoretic models where the pay-off depends on the strategies adopted by each member of the community and the cumulative action of all the members. The suitability of communities for biotechnological applications has been studied using such game-theoretic models. The population dynamics and extracellular enzyme synthesis are modelled, which is then used to predict the nature of the community—competition, cooperation, and co-existence [133].

#### 3.3.4 Agent-based or individual-based modelling of microbial communities

Microbial communities can also be modelled at the ‘individual level’. The growth rate and the specific substrate uptake rate of each individual organism are used to develop agent-based or individual-based models [73]. The properties of the community are defined as the collection of the properties of the individuals in the community, unlike in population-based models, where they are considered to be the cumulative properties of the distinct populations. This approach also addresses the heterogeneity of populations. BacSim and iDynoMics are some tools that can be used to simulate individual-based models of communities [134], [135].

## 4 Designing communities: getting community composition right

The environmental conditions required for a given bioprocess may not always exist in nature, demanding the design of an artificial community based on a defined end goal. The members of the community can be chosen based on their ability to utilise multiple carbon sources or based on their ability to produce specific products with a good yield.

Different design approaches for artificial communities include enrichment, community reduction, combinatorial evaluation, and computational model-based design [37]. Enrichment involves modifications to the environmental conditions that would enhance the growth of a species that is capable of executing a desired function. Community reduction includes a screening process to retain desirable species and excludes undesirable members. Combinatorial evaluation involves evaluating all possible combinations of a set of candidate species for their performance of a desired function and selection of the best communities. Mechanistic models have also been used to evaluate potential community compositions *in silico,* which can be validated further through experiments [37].

Borenstein *et al.* have developed CoMiDA, a method that identifies minimal sets of microbes that can together provide the enzymatic capability to synthesise a set of desired target metabolites from a predefined set of substrates [136]. The method incorporates a graph-theoretic approach and an integer linear programming framework to map out metabolic paths from the substrates to products while simultaneously minimising the number of microbial species required for catalysing these metabolic reactions. MultiPus is another method that minimises the number of reactions and inter-microbial transfers instead of the number of species [137]. MultiPus has been applied to analyse the theoretical production of cephalosporin C and 1,3-propanediol by diverse synthetic microbial communities. NetCooperate [138] is a web-based tool that provides host-microbe and microbe-microbe cooperative potential. It accepts a pair of metabolic networks as input and calculates the ‘Metabolic Complementarity Index’ – a measure of the biosynthetic complementarity of the two organisms. It also provides a list of potential syntrophic metabolites.

Currently, most of the experimental studies deal with artificial communities that are designed based on prior knowledge. Experimental verification of multiple communities is very laborious and time-consuming. A study by Wilken *et al.* has tried to identify the best partners for anaerobic fungi [139]. A limitation of this study is that anaerobic fungi are a fixed member of the community, and only the partner species is identified computationally, and the size of the community is restricted to two. However, if we were to expand the size of the community and the number of organisms to choose from, the problem becomes computationally expensive. As yet, there are only a very few methods to design the community composition. The existing methods often identify the minimal set of microbes but not the most efficient set that is required to achieve a goal, as described above. This asserts the need for better-automated algorithms to analyse all possible communities better and identify the best community for a given purpose.

## 5 Improving community performance: model-driven rational strain design

Once we have a functional microbial community for a process, we can explore further optimisation of the community via strain improvement of the constituent microbes. As discussed earlier, the microbes may not always have sufficient yield and productivity in their native state. Therefore, metabolically engineering these strains to remove genes responsible for the production of unwanted by-products, or over-expressing the genes responsible for the synthesis of the product, can increase the yield significantly.

Similarly, the organisms of a community can also be engineered to cooperate better. This is especially important in artificial communities, where one organism can quickly outgrow the other if it has a greater growth advantage. This scenario can be prevented if the organisms are engineered such that each organism is auxotrophic for a metabolite supplied by its partner. This creates a system of checks and balances, ensuring the stability of the community. The biosynthetic pathway of salidroside has been partly engineered in a co-culture of two *E. coli* strains. The strains were further engineered such that they utilise various carbon sources to reduce substrate competition and ensure cooperation. Also, one strain supports the growth of the other by over-producing the amino acid for which the partner is auxotrophic [140], [141].

A wide range of in silico strain optimisation algorithms exist; these utilise *individual* microbial GEMs as the standard input. One of the pioneering algorithms widely used to predict gene and reaction knock-out strategies is OptKnock [142]. OptKnock computes reaction deletion targets through bi-level mixed-integer linear programming (MILP), which couples objectives of biomass formation with target metabolite production. Knock-out targets predicted by OptKnock have been validated successfully through experiments. Some recent examples include the prediction of gene knock-outs from the non-model yeast *Issatchenkia orientalis* SD108 for overproduction of succinic acid [143]. In a co-culture of *Clostridium autoethanogenum* and *Clostridium kluyveri,* OptKnock has been used to identify gene knock-outs to improve the production of medium-chain fatty-acids [144]. This study shows that strain optimisation algorithms have the potential to be extended to microbial communities.

Other algorithms that have been developed for optimisation of single strains include Flux Scanning based on Enforced Objective Flux (FSEOF) [145] and Flux Variability Scanning based on Enforced Objective Flux (FVSEOF) [146]. These methods identify reactions that should be up-regulated or down-regulated and that are either positively or negatively correlated with the target product synthesis. FSEOF was first introduced to identify amplification targets for increased lycopene production [145]. In a recent study, it was used to predict non-conventional targets such as nucleoside inosine to enhance the production of hyaluronan from recombinant *Lactococcus lactis* [147].

OptGene [148] is an algorithm that uses simulated annealing and evolutionary algorithms to optimise the set of gene knock-outs that maximise a given objective function. OptStrain [149] optimises the organism for non-native functionalities. It identifies the maximum yield path for biotransformation and minimises the number of non-native reactions required to achieve the target yield. EMILiO [150] identifies the optimal flux ranges needed to maximise the yield of the target product. It uses successive linear programming to decrease the time necessary for computation.

Some recent algorithms use alternate approaches to the traditional bilevel optimisation problems. NIHBA [151] is a network interdiction model which uses a hybrid Bender’s algorithm to achieve computational strain design. MODCELL2 [152] is an algorithm that aids modular cell engineering, where each parent strain is converted to a pool of modular cells that can be integrated with exchange modules to arrive at the desired strain. Once the modular cells are designed, they can be assembled in multiple ways to design different strains for different objectives.

OptRAM (Optimisation of Regulatory And Metabolic Networks) [153] can identify amplification and knock-out targets for both genes and transcription factors. It uses the Integrated Deduced Regulation And Metabolism (IDREAM) framework to develop integrated regulatory-metabolic models, which are then optimised for target product synthesis using the OptRAM algorithm. As discussed earlier, the problem is underdetermined and can have multiple solutions for a given objective. All the above algorithms choose one of the solutions randomly. Looking at the range of flux values a reaction can take, can present a better picture of the metabolic capabilities of an organism. OptForce uses FVA to identify the flux variability for the target reaction and optimises the set of gene deletions and/or amplifications that can maximise the upper limit of the flux range for the target reaction.

MEWpy [154] is an integrated strain optimisation platform that can utilise metabolic, enzymatic, and regulatory constraint-based models. This workbench uses the GECKO toolbox and OptRAM algorithms which integrate proteomic and transcriptional regulation for better predictions. Table 3 presents a comprehensive list of computational methods that use GEMs to predict novel over-expression and knock-outs targets to improve the biosynthesis of products.

**Table 3:** Tools for computational strain design. (HI)–Heterologous insertion; (RD)–Reaction deletion; (RA)–Reaction amplification; (RDR)–Reaction down-regulation; (GD)–Gene deletion; (GA)–Gene amplification; (GDR)–Gene down-regulation.

**Algorithm**	**Description**	**Type of intervention**	**Heuristic/Exact**	**Ref.**
OptKnock	It is a bi-level optimisation framework where the inner problem maximises the cellular objective while the outer problem maximises the bioengineering objective. Most of the future algorithms adopt a similar framework.	RD	Exact	[142]
OptStrain	Identifies the non-native reactions to be cloned into the microbe to achieve heterologous functionality.	RD/ HI	Exact	[149]
OptReg	Identifies both reaction deletions and amplifications using OptKnock framework	RD/RA	Exact	[155]
OptORF	Uses GPR association rules to identify gene knock-outs and amplifications. (Penalty for each intervention)	GD/GA	Exact	[156]
OptForce	Compares the flux ranges for wild type and mutant network using FVA and thereby identifies intervention targets.	RD/RA/RDR	Exact	[157]
FSEOF	Scans the changes in the flux distribution of the metabolic network when the product synthesis is pushed. The reactions that show an increase/decrease in flux, as a result, are chosen as potential over-expression/deletion targets	RD/RA	Exact	[145]
EMILiO	Uses iterative linear optimisation to identify the optimal flux values for each intervention target.	Flux value	Exact	[150]
CASOP	Uses elementary modes to identify deletion and overexpression targets.	RD/RA	Exact	[158]
cMCS	Identifies reaction deletions by identifying constrained Minimal Cut-Sets (cMCS), which are MCS that are restricted to maintain certain functionalities. These constraints are chosen such that the bioengineering objective is met.	RD	Exact	[159]
CosMos	Identifies optimal flux value by continuous modification of flux bounds of a reaction	Flux value	Exact	[160]
NIHBA	Uses evolutionary game theory and a hybrid Bender’s algorithm to optimise the strain design	RD	Exact	[151]
OptGene	Uses genetic algorithms to identify knock-out targets	RD	Heuristic	[148]
ModCell2	Uses evolutionary algorithms to achieve modular cell engineering. Here, the parent strain is transformed into a modular cell, and many such exchange modules constitute a strain design	RD	Heuristic	[152]
OptRAM	Uses simulated annealing to identify knock-outs, up-regulation, and down-regulation of genes and transcription factors from the IDREAM integrated network framework	GD/GA/GDR	Heuristic	[153]

Constructing microbial communities of engineered strains can lead to discovering novel phenotypes and provide improved metabolic capabilities for the biosynthesis of industrially relevant chemicals. While most of the methods mentioned above have proven successful in predicting optimisation strategies for single-species metabolic models, algorithms suitable for microbial communities are few and far between. This is an important area of future research, particularly owing to the computational complexity of dealing with large microbial community models composed of a higher number of reactions and metabolites. Thus, there is a need to simplify and specialise such algorithms for use in communities. One must also consider parameters such as the total number of genetic interventions and how many such interventions need to be implemented in each organism in the community.

## 6 Summary and outlook

Microbial communities have been long exploited for fermentation processes such as bread-making and winemaking. Yet, to systematically study, understand, design, and ultimately manipulate these microbial consortia, it is imperative to use mathematical approaches that capture key aspects of microbial metabolism and interactions. Though graph-based, population-based, and other ecological models provide useful insight into the behaviour of microbial communities, constraint-based modelling offers a deeper understanding of microbial metabolism. Despite the strides made in constraint-based modelling over the last two decades, many important challenges need to be surmounted to apply and extend these modelling methods to large community metabolic networks. There is a need for better strategies to formulate the community composition and specialised algorithms for synthetically engineering microbial communities. Advancements in community modelling and strain design can aid the accurate prediction of community phenotypes and thereby establish superior bioprocesses.

Recent advances in genome sequencing techniques have created a massive influx of GEMs. This, in turn, warrants a need for better curation tools as the availability of high-quality GEMs plays a vital role in the metabolic modelling of microbial communities. A major experimental technique that has influenced the development of metabolic models, particularly their validation, is ^13^C-MFA (metabolic flux analysis) [161]. It uses labelled tracers to quantify metabolic fluxes, which enables the construction of a flux map. The application of ^13^C-MFA to multi-species communities is difficult as it requires physical separation of the cells. Gebreselassie and Antoniewicz have proposed a novel approach to perform ^13^C-MFA in communities [162]. However, the ready utilisation of this approach is still challenging as it requires careful selection of the isotopic tracers. Therefore, there is a pressing need for advanced techniques that can simplify the use of ^13^C-MFA to study microbial communities.

In this review, we have extensively dealt with metabolism and flux analysis—*fluxomics*. There are several other omics fields, each of which shines a light on different microbial interactions in communities. The use and integration of other omic data, from transcriptomics, proteomics, and metabolomics, alongside fluxomics, can provide a better, holistic picture of microbial community interactions [163], [164]. Multi-omic data integration can range from simple two-layer [165], [166] to multi-layer integration [167], [168] that can be more computationally demanding. Assembling integrated models for microbial communities can be twice as challenging due to the paucity of multi-omic data and the complexity in data processing [169], [170]. However, it holds tremendous potential to help us understand the functional role of each member of the community and the complex forces at work that result in a specific phenotype. Integrating FBA-based methods with network science-backed models has also been explored and found to provide a better understanding of the structure and function of metabolic networks [171]. Advancements in integrating diverse modelling approaches and omics data can result in accurate and biologically relevant predictions of microbial metabolism, and thereby enable better strain design.

In the recent years, Machine Learning (ML) has impacted nearly every field of science and engineering. ML uses data-driven algorithms that ‘train’ on supervised examples and improve its performance through experience, i.e., learning. ML is relatively nascent in metabolic engineering, yet it holds immense potential [172]. It can be used to identify and annotate protein sequences in genomes [173], promoter design [174], and process control and optimisation [175]. It is instrumental in the quantitative prediction of metabolic fluxes. A significant bottleneck in implementing ML techniques though, is the non-availability of large datasets with high reproducibility. Generation of high-quality high-throughput data for microbial communities and integration of ML-based approaches can bolster the use of microbial communities for bioproduction.

Through this review, we have presented a broad overview of many mathematical methods, especially those based on the paradigm of constraint-based modelling, which is quite versatile and has been successfully employed to improve our understanding of microbial communities. Despite the significant advances documented in this review, numerous unsolved problems remain, as the field promises to be a seedbed of active research over the coming years.

## 7 Author agreement statement

We the undersigned declare that this manuscript is original, has not been published before and is not currently being considered for publication elsewhere. We confirm that the manuscript has been read and approved by all named authors and that there are no other persons who satisfied the criteria for authorship but are not listed. We further confirm that the order of authors listed in the manuscript has been approved by all of us. We understand that the Corresponding Author is the sole contact for the Editorial process. He/she is responsible for communicating with the other authors about progress, submissions of revisions and final approval of proofs.

## CRediT authorship contribution statement

**Maziya Ibrahim:** Conceptualization, Writing - original draft. **Lavanya Raajaraam:** Conceptualization, Writing - original draft. **Karthik Raman:** Conceptualization.

## Declaration of Competing Interest

The authors declare that they have no known competing financial interests or personal relationships that could have appeared to influence the work reported in this paper.
